# Cognitive Recovery After Out‐of‐Hospital Cardiac Arrest: Insights Into Improvement Over 6 Months and the Role of Arrest Duration

**DOI:** 10.1161/JAHA.125.042637

**Published:** 2025-11-03

**Authors:** Jeanet A. E. Brouer, Lisa G. Oestergaard, Hans Eiskjær, John Bro‐Jeppesen, Lola Qvist Kristensen

**Affiliations:** ^1^ Department of Physiotherapy and Occupational Therapy Aarhus University Hospital Aarhus Denmark; ^2^ DEFACTUM Central Denmark Region Aarhus Denmark; ^3^ Faculty of Health, Department of Public Health Aarhus University Aarhus Denmark; ^4^ Department of Cardiology Aarhus University Hospital Aarhus Denmark; ^5^ Department of Clinical Medicine Aarhus University Aarhus Denmark

**Keywords:** cognitive function, cognitive recovery, duration of cardiac arrest, Montreal Cognitive Assessment (MoCA), out‐of‐hospital cardiac arrest, Cardiovascular Disease, Risk Factors

## Abstract

**Background:**

Out‐of‐hospital cardiac arrest is a significant cause of mortality and morbidity worldwide. Although resuscitation advancements have increased survival, many survivors suffer cognitive impairments that affect their quality of life. Most research has focused on neurological outcomes, whereas little attention has been paid to cognitive function. The aim is to investigate the proportion of cognitive impairment in survivors of out‐of‐hospital cardiac arrest at discharge and 6 months after cardiac arrest and to investigate the association between the duration of cardiac arrest and level of cognitive function.

**Methods:**

In this prospective cohort study, 184 survivors of out‐of‐hospital cardiac arrest were assessed using the Montreal Cognitive Assessment screening tool. Duration of cardiac arrest was defined by no flow, low flow, and time to return of spontaneous circulation. Multiple logistic regression analysis provided odds ratios (OR) and CI.

**Results:**

Of 184 survivors assessed, 26% had normal cognitive function at discharge, increasing to 67% at 6 months (n=149). Median Montreal Cognitive Assessment score improved from 23 (Q25–Q75: 20–26) to 26 (Q25–Q75: 24–28). At 6 months, adjusted ORs per minute were 1.03 (95% CI, 1.00–1.07) for low flow and 1.03 (95% CI, 1.00–1.07) for time to return of spontaneous circulation, whereas associations at discharge were near null (aOR≈0.99). No significant association was found either at discharge or follow‐up.

**Conclusions:**

Cognitive function improved considerably within 6 months following cardiac arrest, with the proportion of patients exhibiting normal cognitive function increasing from 26% to 67%. This study found no association between the duration of cardiac arrest and cognitive function.

**Nonstandard Abbreviations and Acronyms**
MoCA: Montreal Cognitive Assessment
OHCA: out‐of‐hospital cardiac arrest
ROSC: return of spontaneous circulation

**Clinical Perspective**

**What Is New?**

- Cognitive recovery after out‐of‐hospital cardiac arrest is substantial, with two thirds of patients showing normal function at 6 months.
- No association was found between the duration of cardiac arrest and cognitive function when assessed with the Montreal Cognitive Assessment.

**What Are the Clinical Implications?**

- Cognitive screening with the Montreal Cognitive Assessment should be part of routine postresuscitation care to identify impairments early.
- Clinicians can provide more optimistic counseling to survivors and families, as cognitive improvement is common despite prolonged arrest duration.
- Rehabilitation strategies should focus on memory and executive domains, which are the most impaired but also show the greatest recovery potential.

Out‐of‐hospital cardiac arrest (OHCA) is a major cause of morbidity and mortality worldwide; however, survival rates have improved over the past 2 decades.^1^, ^2^ In Denmark, the 30‐day survival rate rose from 4% in 2001 to 14.4% in 2023; worldwide, the 30‐day survival ranges from 3% to 18%.^3^, ^4^, ^5^ This increase can be attributed to the widespread implementation of prehospital cardiopulmonary resuscitation (CPR) training and increased public access to automated external defibrillators,^6^ along with advancements in postresuscitation care following hospital admission.^7^, ^8^

Hypoxic brain injury is a common sequela of cardiac arrest,^9^, ^10^, ^11^, ^12^ often leading to cognitive impairments ranging from mild to severe.^13^, ^14^, ^15^ These impairments occur primarily in the domains of memory, attention, and executive function, depending on the region of injury.^16^, ^17^

Most resuscitation science has focused on survival and survival with favorable neurological outcomes, typically assessed using the 5‐point Cerebral Performance Category scale or the 6‐point modified Rankin Scale. However, knowledge is limited about the specific cognitive impairments commonly observed among survivors of OHCA, which may have significant implications on daily activities. Previous studies have explored the association between the duration of cardiac arrest and the neurological outcomes in survivors of OHCA; however, they have not specifically focused on cognitive function.^18^, ^19^, ^20^, ^21^

The duration of the cardiac arrest is widely recognized as an important factor influencing survival and neurological outcomes following OHCA. A cohort study (N=11 368) demonstrated a steady decline in the likelihood of survival with a favorable neurological outcome (modified Rankin Scale score ≤3) at hospital discharge as the duration of professional CPR increased.^18^

The duration of cardiac arrest can be defined by 2 distinct intervals: (1) no flow (interval from collapse to initiation of CPR) and (2) low flow (interval from start of CPR to return of spontaneous circulation [ROSC] or termination of resuscitation).^19^, ^22^ Despite a strong association between the duration of OHCA and an adverse neurological outcome, complete recovery after prolonged CPR may be achieved in selected cases.^23^, ^24^

Research indicates that approximately 50% of survivors of OHCA achieve a Cerebral Performance Category score of <2, which is internationally recognized as a successful outcome. However, almost half of these survivors later experience cognitive impairments, highlighting the complexity and challenges in accurately evaluating long‐term outcomes.^16^, ^17^

Limited knowledge exists regarding the relationship between the duration of cardiac arrest and cognitive function, especially when assessed using the Montreal Cognitive Assessment (MoCA) screening tool.

This study aimed to investigate the proportion of cognitive impairment at hospital discharge and its progression over 6 months following cardiac arrest. Additionally, it sought to investigate the association between the duration of cardiac arrest and level of cognitive function at both hospital discharge and 6‐month follow‐up in a population of survivors of OHCA. We hypothesized that a longer duration of cardiac arrest would be associated with poorer cognitive outcomes.

## METHODS

The data that support the findings of this study are not publicly available due to Danish and European data protection regulations concerning sensitive personal health information. The statistical code and detailed descriptions of the analytic methods can be made available upon request to the corresponding author.

### Design, Setting, and Participants

This study was a prospective single‐center observational cohort study with a follow‐up period of 6 months after cardiac arrest. The study was conducted as a substudy of an ongoing cohort study investigating activities of daily living after surviving cardiac arrest.^25^, ^26^ The present study uses data on the duration of cardiac arrest and cognitive function collected from this more extensive study. Patients were recruited from the Department of Cardiology at Aarhus University Hospital, Denmark, between June 2019 and March 2023. Aarhus University Hospital is a tertiary cardiac center and the centralized admission site for patients with OHCA from the entire Central Denmark Region, which comprises 19 municipalities and has a population of 1.4 million.

**Figure 1:**
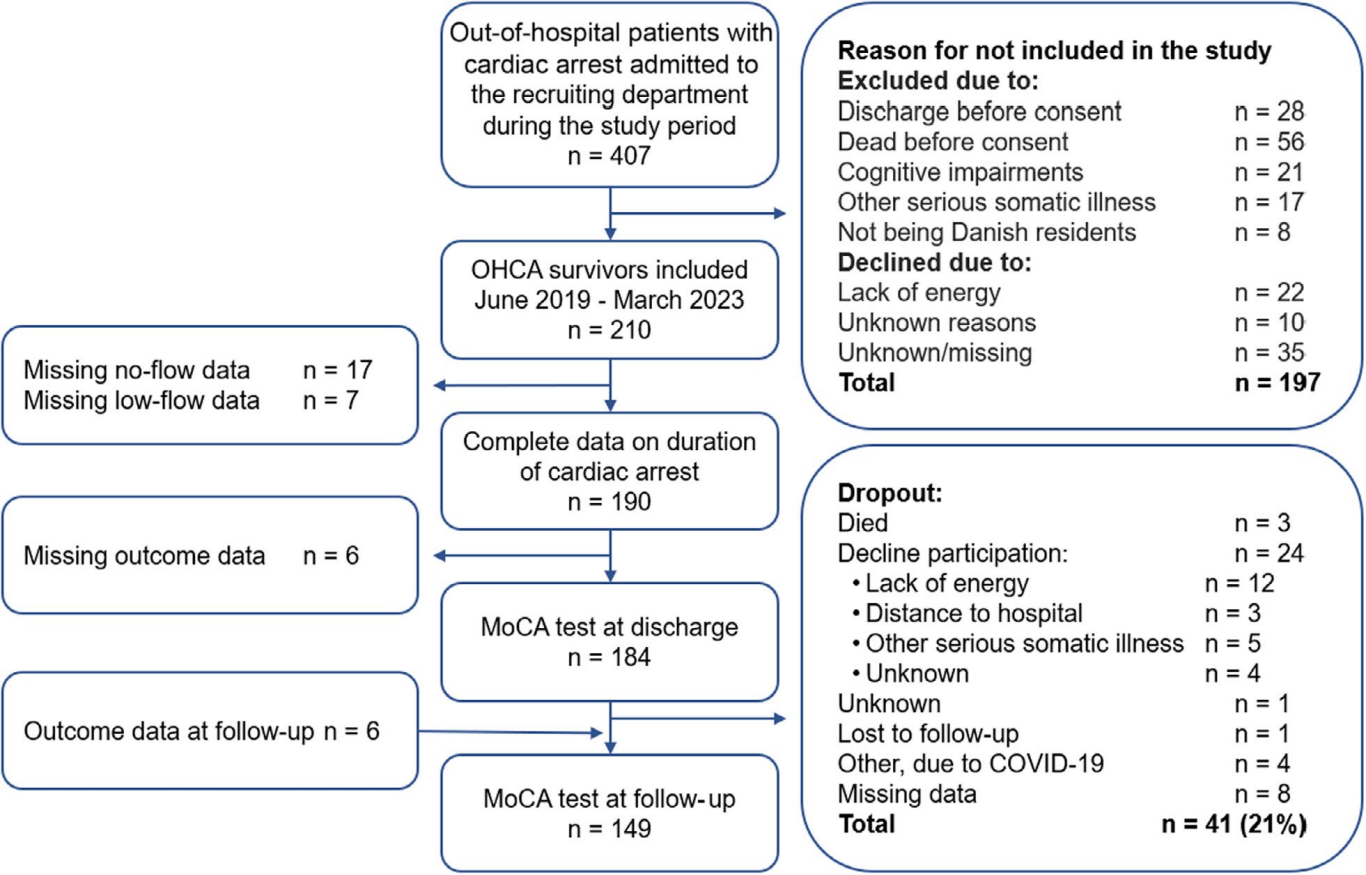
Flow chart of participation in the study. MoCA indicates Montreal Cognitive Assessment; and OHCA, out‐of‐hospital cardiac arrest.

Data were collected after verbal and written consent had been obtained. Inclusion criteria were successful resuscitation from OHCA, age ≥18 years, and sufficient command of the Danish language to understand participant information and to give informed consent, based on the occupational therapist’s assessment. Participants were excluded if no data were available on the duration of cardiac arrest or the assessment of cognitive function (Figure 1). The study was registered at the Central Denmark Region (1‐16‐02‐261‐19). Additionally, the Ethics Committee waived the need for further registration of the study (1‐10‐72‐1‐20). Furthermore, this study was conducted in accordance with the Strengthening the Reporting of Observational Studies in Epidemiology statement.^27^

### Data Collection

#### Demographics and Clinical Variables

Sociodemographic data, such as marital status, education, and employment status, were self‐reported. Data on age, sex, comorbidities, witnessed arrest, bystander CPR, primary rhythm, and location were extracted from the Electronic Patient Journal and the Central Prehospital Patient Journal from Aarhus University Hospital, Denmark, at baseline.

#### Duration of Cardiac Arrest

The primary independent variable of interest was the duration of cardiac arrest measured by no flow and low flow. No flow was defined as the time interval in minutes from the bystander emergency call (recorded when the call was answered at the emergency services and assumed to be the approximate arrest time) to the CPR initiation. Low‐flow duration was defined as the time in minutes from the start of CPR to ROSC. Data are recorded and documented in the national prehospital database using standardized procedures by trained emergency personnel, ensuring consistency and structure in the estimate. The total duration until ROSC was achieved was calculated by adding no‐flow and low‐flow times. Data were collected from the Electronic Patient Journal and the Prehospital Patient Journal, which were reported by the prehospital emergency physician.

#### The Montreal Cognitive Assessment

Cognitive function was assessed within the past 5 days before discharge from the hospital and repeated at approximately 6 months (±14 days) after cardiac arrest, both assessed by the MoCA tool.

The MoCA is a widely used screening tool for mild cognitive impairment. It assesses 7 cognitive domains: visuospatial and executive abilities, naming, attention, language, abstraction, delayed recall, and orientation.^28^ Each cognitive domain is assigned a specific score, which is summed to produce a total score ranging from 0 to 30 points. The test can be administered in approximately 10 to 15 minutes and is a brief paper‐and‐pencil cognitive screening tool. The MoCA score is adjusted for educational level by awarding 1 extra point to anyone with ≤12 years of formal education. This adjustment aims to minimize the risk of classifying less educated subjects as cognitively impaired. A cutoff of ≥26 is considered normal cognitive function.^28^ Both baseline and follow‐up assessments were conducted by 1 of 4 certified and experienced occupational therapists at the Department of Cardiology, Aarhus University Hospital.

### Statistical Analysis

Descriptive statistics were used to present participants’ baseline characteristics. The distribution of data was presented by frequencies (n) and proportions (%) for categorical variables and by means±SD for continuous variables if normally distributed or otherwise as medians with quartiles (Q25–Q75). The distribution of continuous variables was assessed visually using histograms and Q–Q plots.

The analysis was performed by logistic regression, calculating odds ratio (OR) and 95% CI to estimate the odds of a cognitive function below the cutoff (<26) on MoCA after cardiac arrest. An odds ratio ∼2 was considered clinically meaningful in this context, despite the *P* value. This effect size—indicating a doubling of the odds of MoCA <26—reflects a meaningful difference in cognitive function. Secondarily, the association between the duration of cardiac arrest and cognitive function after 6 months was examined. We assessed potential nonlinear relationships, by examining arrest duration variables (low flow and no flow) using quadratic terms in the logistic regression models. Additionally, we evaluated interactions between arrest duration and demographic variables age and sex. We evaluated standard logistic regression assumptions: independence of observations, linearity of covariates and multicollinearity (pairwise correlations, variance inflation factors). Model fit was assessed by calibration (Hosmer–Lemeshow test) and discrimination (area under the receiver operating characteristic curve).

Both crude and adjusted ORs were calculated for the associations. We adjusted for potential confounders, including age, sex, comorbidities, and cardiac arrest characteristics (witnessed arrest, bystander CPR, initial rhythm, and arrest location). These variables were available in the data set and are recognized in the literature as important determinants of postarrest neurological and cognitive outcomes.^5^, ^20^ They may confound the association between arrest duration and cognitive function through, for example, age‐related vulnerability, sex‐related differences in outcome or the impact of comorbidities on cerebral resilience. A *P* value of <0.05 was considered statistically significant.

Missing follow‐up was largely explained by practical or health problems, and baseline MoCA scores indicated no systematic association with cognitive function, supporting the missing at random assumptions. We conducted a sensitivity analysis of MoCA score progression using imputed data to address missing values at baseline (n=6) and follow‐up (n=38). Missingness was assumed to be missing at random, except for 3 individuals who died before follow‐up and therefore were excluded from the imputation model at follow‐up. Multiple imputation by chained equations with 30 imputations was applied to ensure robust estimation. The model included complete baseline data, and, both baseline and follow‐up MoCA scores were used to inform the imputations.^29^, ^30^ Supplementary stratified analyses by age and comorbidity burden were performed to explore potential subgroup differences, with results presented in the Data S1.

All analyses and data management were conducted in STATA version 18.0 (StataCorp, College Station, TX, USA).

## RESULTS

### Study Population

Among admitted survivors after OHCA, who were alive at hospital discharge, 210 met the inclusion criteria. As shown in the flow chart (Figure 1), data on the duration of cardiac arrest were available for a study population of 190 participants. Of those, MoCA scores were available for 184 participants. Data regarding MoCA were available at follow‐up in 149 participants. At follow‐up, a dropout rate of 21% was observed, with the majority citing lack of energy as the primary reason for declining participation (Figure 1).

Overall patient characteristics are shown in Table 1. The participants’ mean age was 63 years (SD ±13), with 83% being male. The proportion of witnessed arrests was 96%, and 91% received bystander CPR. Ventricular fibrillation/ventricular tachycardia was the initial rhythm in 86% of the participants. Regarding marital status, 74% were living with a spouse. Retirees accounted for 47%, and 41% were employed full ime before their cardiac arrest. Cardiovascular comorbidities were present in 44%. Notably, 88% of the participants were referred for rehabilitation upon discharge.

**Table 1:** Characteristics of Survivors of OHCA at Discharge and 6‐Month Follow‐Up and the Baseline Score of Those Who Dropped Out

Demographics	Baseline	Follow‐up	Dropout
	N=190	N=149	N=41
Age, y, mean±SD	63±13	62±14	68±12
Sex, n (%)			
Male	157 (83)	121 (81)	36 (88)
Female	33 (17)	28 (19)	5 (12)
Cardiac arrest variables, n (%)			
Witnessed arrest	182 (96)	142 (96)	40 (98)
Bystander cardiopulmonary resuscitation	170 (91)	133 (90)	37 (93)
Initial rhythm			
Ventricular fibrillation/ventricular tachycardia	160 (86)	134 (92)	26 (65)
Pulseless electrical activity	9 (5)	5 (3)	4 (10)
Asystole	9 (5)	4 (3)	5 (13)
Location			
Public	90 (48)	70 (48)	20 (49)
Home	93 (50)	73 (50)	20 (49)
Sociodemographic variables			
Marital status, n (%)			
Living alone	43 (23)	35 (23)	8 (20)
Living with spouse	141 (74)	111 (75)	30 (73)
Living with other	6 (3)	3 (2)	3 (7)
Education, n (%)			
≤ 9 y	32 (17)	23 (15)	9 (22)
10–12 y	85 (45)	65 (44)	20 (49)
13–15 y	42 (22)	36 (24)	6 (15)
≥ 16 y	20 (11)	16 (11)	4 (10)
Unknown	11 (6)	9 (6)	2 (5)
Employment status, n (%)			
Full time (≥37 h/wk)	77 (41)	65 (44)	12 (29)
Part time (<37 h/wk)	14 (7)	10 (7)	4 (10)
Unemployed	6 (3)	6 (4)	
Retired	89 (47)	64 (43)	25 (61)
Under education	3 (2)	3 (2)	
Unknown	1 (0.5)	1 (0.5)	
Medical history			
Comorbidity areas, n (%)			
Cardiovascular	84 (44)	62 (42)	22 (54)
Respiratory	14 (7)	7 (5)	7 (17)
Diabetes	13 (7)	11 (7)	2 (5)
Other medical	29 (15)	18 (12)	11 (27)
Neurologic	18 (9)	12 (8)	6 (15)
Orthopedic/musculoskeletal	19 (10)	14 (9)	5 (12)
Psychiatric	5 (3)	4 (3)	1 (2)
Other	11 (6)	9 (6)	2 (5)
Number of comorbidity areas, n (%)			
None	52 (27)	46 (31)	6 (15)
1 comorbidity	97 (51)	75 (50)	22 (54)
2–4 comorbidities	41 (21)	28 (19)	13 (32)
Referred for rehabilitation	167 (88)	133 (89)	34 (83)
Rehabilitation intervention referrals, n (%)			
Specialized cardiac rehabilitation	56 (29)	50 (34)	6 (15)
Specialized neurorehabilitation	33 (17)	31 (21)	2 (5)
Physical and cognitive rehabilitation in municipality	117 (62)	87 (58)	30 (73)
Duration of cardiac arrest, median (Q25–Q75)			
No flow, min	0 (0–2)	0 (0–2)	0 (0–2)
Low flow, min	12 (7–18)	13 (8–20)	10 (6–15)
Time to return of spontaneous circulation, min	13 (9–20)	14 (10–20)	11 (7–18)
D from cardiac arrest to baseline assessment of Montreal Cognitive Assessment, mean±SD	13±15	14±17	11±9

OHCA indicates out‐of‐hospital cardiac arrest.

Of those who dropped out, the mean age was slightly higher, and most were men. There was a higher prevalence of initial rhythm pulseless electrical activity or asystole. In addition, a higher percentage were retirees and had multiple comorbidities. They were also more likely to have been referred to rehabilitation in the municipality.

Data on no‐flow and low‐flow time were available for 190 participants. The median of no‐flow time was 0 minutes (0–2) with a range of 0 to 10 minutes; the median observed low‐flow was 12 minutes (7–18) with a range of 1 to 102 minutes; and the median of time to ROSC was 13 minutes (9–20).

### Cognitive Function at Discharge and Development Over 6 Months

Table 2 shows the cognitive function at discharge (n=184) and temporal changes over the course of the next 6 months (n=149) after cardiac arrest in survivors of OHCA. A total of 26% had normal cognitive function at hospital discharge, indicated by a MoCA ≥26 score. The median total MoCA score at discharge was 23 (20–26), with scores ranging from 8 to 30 points. Most patients (59%) had a mildly reduced MoCA score (Table 2).

**Table 2:** Prevalence of Cognitive Impairment at Discharge and 6‐Month Follow‐Up and the Baseline Score of Those Who Dropped Out, Measured by MoCA and Divided Into 4 Groups

Cognitive function	Baseline	Follow‐up	Dropout
	N=184	N=149	N=41
Severe 0–9, n (%)	2 (1)	0 (0)	
Moderate 10–17, n (%)	25 (14)	6 (4)	7 (17)
Mild 18–26, n (%)	108 (59)	44 (29)	29 (71)
Normal 26–30, n (%)	49 (26)	99 (67)	5 (12)
Total MoCA score, median (Q25–Q75)	23 (20–26)	26 (24–28)	22 (20–24)

MoCA indicates Montreal Cognitive Assessment.

Of the 41 participants who dropped out, 36 (88%) had a MoCA score <26 at discharge.

At the 6‐month follow‐up, 67% of the participants had normal cognitive function.

The median MoCA score was 26 (24–28), with a range from 12 to 30. This just met the threshold for normal cognitive function (Table 2). Only 4% of patients had moderately impaired cognitive function and none had a severely impaired MoCA score.

**Figure 2:**
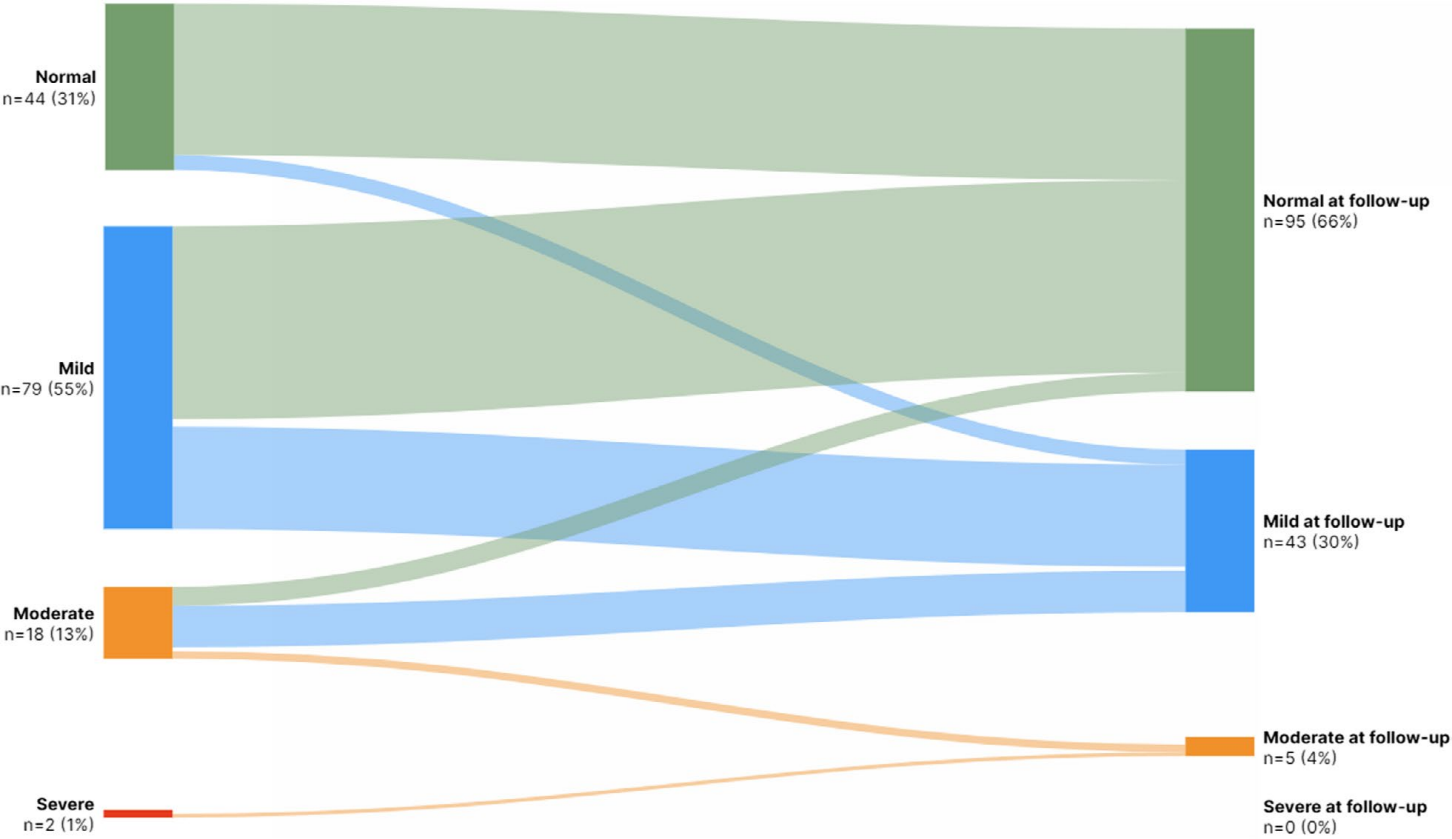
Changes in MoCA between discharge and follow‐up 6 months after cardiac arrest for those where both measurements were present (n=143). MoCA score is divided into 4 groups at baseline and 6 mo after cardiac arrest: Green: 26–30 = normal cognitive function. Blue: 18–25 = mild cognitive impairment. Orange: 10–17 = moderate cognitive impairment. Red: 0–9 = severe cognitive impairment. MoCA indicates Montreal Cognitive Assessment.

Figure 2 presents a Sankey diagram illustrating the distribution of MoCA scores across 4 severity levels at baseline and 6 months after cardiac arrest, illustrating changes in cognitive function over time. Complete MoCA data at both discharge and the 6‐month follow‐up were available for 143 participants.

After 6 months, the proportion of OHCA survivors with normal cognitive function (MoCA ≥26) increased significantly, from 31% at discharge to 67%. Approximately one third of survivors had mild cognitive impairment at the 6‐month follow‐up. The majority of patients (n=18/20, 90%) with moderate or severe cognitive impairment at discharge demonstrated improved cognitive status at 6 months.

**Figure 3:**
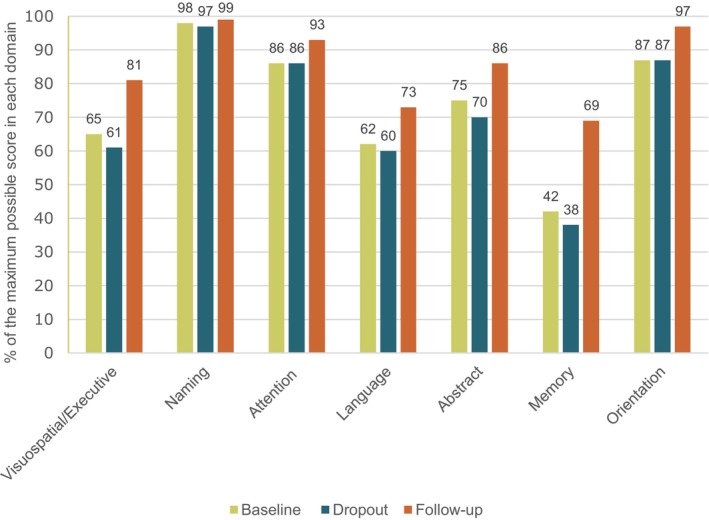
MoCA score among different cognitive domains at hospital discharge, dropout, and 6 months after cardiac arrest. MoCA indicates Montreal Cognitive Assessment.

Figure 3 reveals the 7 cognitive MoCA domains, each with its percentage of the maximum possible score in each domain. An increase in scores is seen at 6 months of follow‐up. Among the cognitive domains assessed, memory was the lowest‐scoring domain for participants, and naming, attention, and orientation had the highest scores. Among those who dropped out, slightly lower scores were observed across all domains. The most notable improvement at 6 months follow‐up were in memory and visuospatial/executive domains.

### Association Between Duration of Cardiac Arrest and Cognitive Function at Hospital Discharge

We found no association between the duration of cardiac arrest and cognitive function at hospital discharge (n=184). The association between the duration of cardiac arrest and cognitive function did not change when adjusting for age, sex, comorbidities, witnessed arrest, bystander CPR, the initial recorded rhythm, and location of arrest, Tables 3 and 4.

**Table 3:** Logistic Regression of the Association Between No Flow and Low Flow (Duration of Cardiac Arrest) and Cognitive Function Measured with MoCA <26 at Hospital Discharge

No.=184	Crude			Adjusted		
	OR	95% CI	*P* value	OR	95% CI	*P* value
No flow	1.12	0.91–1.37	0.286	0.83	0.61–1.13	0.241
Low flow	0.99	0.96–1.02	0.561	0.99	0.96–1.03	0.739
Time to return of spontaneous circulation	0.99	0.97–1.02	0.683	0.99	0.96–1.03	0.654

Adjusted for age, sex, comorbidities (yes/no), witnessed arrest, bystander cardiopulmonary resuscitation, the initial recorded rhythm, and location of cardiac arrest. MoCA indicates Montreal Cognitive Assessment; and OR, odds ratio.

**Table 4:** Sensitivity Analysis Using Logistic Regression of the Association Between No Flow and Low Flow (Duration of Cardiac Arrest) and Cognitive Function Measured With MoCA <26 at Hospital Discharge With Imputed Data

No.=190	Crude			Adjusted		
	OR	95% CI	*P* value	OR	95% CI	*P* value
No flow	1.11	0.91–1.36	0.336	0.92	0.69–1.21	0.550
Low flow	0.99	0.96–1.02	0.567	0.99	0.97–1.03	0.920
Time to return of spontaneous circulation	0.99	0.97–1.02	0.684	0.99	0.97–1.03	0.866

Adjusted for age, sex, comorbidities (yes/no), witnessed arrest, bystander cardiopulmonary resuscitation, the initial recorded rhythm, and location of cardiac arrest. MoCA indicates Montreal Cognitive Assessment; and OR, odds ratio.

The association between no flow and cognitive function at hospital discharge showed an unadjusted OR of 1.12 (95% CI, 0.91–1.37) and an adjusted OR of 0.83 (95% CI, 0.61–1.13). Similarly, the association between low‐flow duration and cognitive function revealed an unadjusted OR of 0.99 (95% CI, 0.96–1.02) and an adjusted OR of 0.99 (95% CI, 0.96–1.03). The association between the total duration until ROSC and cognitive function showed an unadjusted OR of 0.99 (95% CI, 0.97 –1.02) and an adjusted OR of 0.99 (95% CI, 0.96–1.03). None of the results were statistically significant (Tables 3 and 4).

### Association Between Duration of Cardiac Arrest and Cognitive Function After 6 Months

Tables 5 and 6 presents the results of the analysis, revealing that among survivors of OHCA, no association was observed between the duration of cardiac arrest and subsequent cognitive function at 6 months after cardiac arrest (n=149). Adjusting for age, sex, comorbidities, witnessed arrest, bystander CPR, the initial recorded rhythm, and location of arrest did not change this result.

**Table 5:** Logistic Regression of the Association Between No Flow and Low Flow (Duration of Cardiac Arrest) and Cognitive Function Measured With MoCA <26 6 Months After Cardiac Arrest

No.=149	Crude			Adjusted		
	OR	95% CI	*P* value	OR	95% CI	*P* value
No flow	1.03	0.85–1.24	0.797	0.91	0.68–1.23	0.545
Low flow	1.02	0.99–1.05	0.245	1.03	1.00–1.07	0.068
Time to return of spontaneous circulation	1.02	0.99–1.05	0.232	1.03	1.00–1.07	0.065

Adjusted for age, sex, comorbidities (yes/no), witnessed arrest, bystander cardiopulmonary resuscitation, the initial recorded rhythm, and location of cardiac arrest. MoCA indicates Montreal Cognitive Assessment; and OR, odds ratio.

**Table 6:** Sensitivity Analysis Using Logistic Regression of the Association Between No Flow and Low Flow (Duration of Cardiac Arrest) and Cognitive Function Measured With MoCA <26 6 Months After Cardiac Arrest With Imputed Data

No.=187*	Crude			Adjusted		
	OR	95% CI	*P* value	OR	95% CI	*P* value
No flow	1.01	0.84–1.22	0.920	0.95	0.74–1.22	0.708
Low flow	1.01	0.98–1.04	0.442	1.02	0.99–1.06	0.145
Time to return of spontaneous circulation	1.01	0.98–1.04	0.430	1.02	0.99–1.05	0.162

Adjusted for age, sex, comorbidities (yes/no), witnessed arrest, bystander CPR, the initial recorded rhythm and location of cardiac arrest. MoCA indicates Montreal Cognitive Assessment; and OR, odds ratio.

* Three participants died before follow‐up and are not included in the data imputation at follow‐up.

The association between no flow and cognitive function at discharge showed an unadjusted OR of 1.03 (95% CI, 0.85–1.24) and an adjusted OR of 0.91 (95% CI, 0.68–1.23). For low‐flow duration, the unadjusted OR was 1.02 (95% CI, 0.99–1.05), and the adjusted OR was 1.03 (95% CI, 1.00–1.07). Similarly, the association between total duration until ROSC was achieved and cognitive function had an unadjusted OR of 1.02 (95% CI, 0.99–1.05) and an adjusted OR of 1.03 (95% CI, 1.00–1.07). None of the results were statistically significant.

### Robustness of Results

The assumptions of logistic regression modeling including independence of observations, linearity of covariates, and multicollinearity were fulfilled.

Sensitivity analyses using the imputed data sets produced results consistent with the primary analyses and did not alter the overall conclusions (Tables 3 through 6). Stratified analyses by age and comorbidity burden did not reveal consistent differences in the association between duration of cardiac arrest and cognitive function at 6 months (see Data S1). No statistically significant associations were observed in any subgroup.

## DISCUSSION

This study investigated 184 adult survivors of OHCA, revealing that 26% had normal cognitive function at hospital discharge. Six months after cardiac arrest, 67% of the 149 participants showed normal cognitive function (MoCA score >26). Concurrently, the prevalence of severe and moderate cognitive impairments decreased, with the highest improvements being observed in the domains of visuospatial/executive and memory. These findings indicate an improvement in cognitive function among survivors of OHCA from discharge to the 6‐month follow‐up.

Contrary to our study hypothesis, our findings revealed no statistically significant association between the duration of cardiac arrest and the level of cognitive function at either hospital discharge or 6 months after cardiac arrest in patients surviving hospital discharge.

To our knowledge, this is the first study to investigate the association between the duration of cardiac arrest and cognitive function using the MoCA. Previous research has focused on survival and neurological outcomes, measured with Cerebral Performance Category or modified Rankin Scale, rather than cognitive function specifically.^12^, ^29^ The high proportion of survivors of OHCA who subsequently experienced cognitive impairment emphasizes the importance of assessing cognitive function separately from overall neurological function in survivors of OHCA. The present study aimed to address this gap in the literature by examining cognitive function using MoCA.

The findings of this study differ in certain aspects from previous neurologically focused research. Reynolds et al. reported a decline in favorable neurological outcomes at hospital discharge with increasing CPR duration; however, they also noted a relatively high incidence of complete recovery after prolonged CPR.^18^ In contrast, Mion et al. found no association between the duration of cardiac arrest and cognitive impairment at discharge.^30^

Differences in study populations and in how the studies define the duration of cardiac arrest may explain some of these discrepancies. For instance, Guy et al.’s study of over 7000 OHCA cases demonstrated that no‐flow duration significantly affects the likelihood of favorable OHCA outcomes at the time of hospital discharge in North America.^20^ They found that for each additional minute of no flow, the adjusted risk of a favorable neurologic outcome decreased by 13%. Notably, no patients with a no‐flow duration >20 minutes achieved a favorable neurological outcome. To ensure the validity of no‐flow estimates, they excluded patients if the arrest was unwitnessed or if CPR or a defibrillator was administered by bystanders. In contrast, our study did not exclude unwitnessed arrests or differentiate between bystander and emergency medical personnel‐administered CPR. Additionally, participants were included only after being transferred to the cardiology ward, regardless of their prior stay in the intensive care unit. This may have led to a selected population in our study.

The duration of cardiac arrest in OHCA can be challenging to assess and is often subject to a degree of imprecision.^20^ The accuracy and reliability of witnesses’ recollection of event times can vary, which can further influence the reported no‐flow and low‐flow durations.^18^ In Denmark, however, the prehospital system systematically records witness calls and the duration of witness CPR. Furthermore, factors such as age and preexisting health conditions can influence how patients tolerate low‐flow conditions, which may affect the outcome.^11^

### Clinical Impact

The findings of this study have important implications for clinical practice. Our findings indicate that many patients experience cognitive recovery following a cardiac arrest, as measured by MoCA. Consequently, optimism about potential cognitive recovery can be maintained, and efforts to optimize patient care should focus on early identification and intervention for cognitive impairments.^11^, ^26^

Routine cognitive screening of survivors of OHCA using MoCA is recommended by the European Resuscitation Council and the European Society of Intensive Care Medicine to detect early signs of cognitive impairment.^11^ MoCA has proven to be a valuable tool in this context, with a cutoff score of 26 providing a reliable indicator of cognitive impairment.^28^ The test has demonstrated high sensitivity (90%) and specificity (87%) for identifying mild cognitive impairment, offering clinicians confidence in its utility.^28^, ^31^, ^32^ Moreover, research has shown that MoCA has a strong construct validity^33^ and good internal consistency (Cronbach’s alpha=0.83), indicating its reliability^28^ and accuracy in measuring cognitive impairment.

Screening should ideally occur both before discharge and at follow‐up to observe long‐term cognitive recovery.^11^ Early identification of cognitive impairment is essential as it allows for referral to a targeted cognitive rehabilitation programme.^26^ Figure 3 shows that, at 6 months, the greatest cognitive improvements were observed in visuospatial/executive functions and memory, followed by improvements in abstract thinking and language. These insights may inform rehabilitation strategies, such as memory training, concentration exercises and strategies for managing cognitive fatigue. However, the clinical significance of individual MoCA domains remains uncertain.

As survival rates improve, cognitive outcomes are becoming an increasingly critical component of postresuscitation care and a key factor in restoring daily life activities. Early identification of cognitive impairments may support clinicians in tailoring rehabilitation interventions to address specific deficits, with the potential to improve everyday quality of life after discharge. The use of MoCA provides a standardized and validated approach to cognitive assessment, supported by evidence of its sensitivity, specificity, and predictive values. However, it has limitations, particularly in the assessment of processing speed. Combining the MoCA with more sensitive tools, such as the Symbol Digit Modalities Test, may offer a more comprehensive cognitive assessments in future research or clinical practice.^31^

### Strengths and Limitations

A key strength of our study is its prospective design, allowing for cognitive assessment at 2 time points: hospital discharge (n=184) and 6 months after cardiac arrest (n=149). This longitudinal approach captures both immediate and longer‐term cognitive outcomes. Furthermore, MoCA provides a detailed evaluation across cognitive domains, enhancing our understanding of specific impairments in survivors of OHCA.^7^, ^28^

In Denmark, prehospital care systematically documents no‐flow and low‐flow times, including cases where resuscitation is initiated by witnesses. All data are validated in the prehospital database to ensure high data quality. The fact that data on no‐flow times were recorded using standardized procedures by trained emergency personnel, ensured consistency and structure in the estimates provided in this study. Additionally, Denmark places a strong emphasis on promoting bystander intervention during cardiac arrests, as early bystander CPR can significantly improve survival rates. CPR training has been provided in schools and workplaces, and many individuals have registered as volunteer first responders (“heart runners”). This effort has led to a high percentage of cases where CPR is initiated immediately by witnesses, resulting in minimal no‐flow time. In our study population, 61% of cases had 0 minutes of no of OHCA flow, indicating that CPR was initiated immediately.

However, the study also has limitations.

Many participants had 0 no‐flow time, likely due to high public awareness and prompt resuscitation efforts in Denmark. In addition, inclusion occurred only after transfer to a general ward, indicating that participants were clinically stable. This may introduce selection bias and limit generalizability.

Another limitation is the lack of information on the participants’ actual engagement with the rehabilitation service to which they were referred. Although referrals included cardiac, neurorehabilitation, or municipal cognitive rehabilitation, the specific content and degree of participation remain unknown. This limits our ability to assess how postdischarge rehabilitation may have influenced cognitive outcomes.

Although we adjusted for well‐established potential confounders, such as age, sex, comorbidities, and witnessed arrest, residual confounding might still have influenced the results. Factors such as inpatient interventions or other unmeasured variables could also play a role. An additional limitation is the lack of data on postresuscitation care, such as targeted temperature management, sedation, and intensive care interventions, all of which are known to affect outcomes after cardiac arrest.^11^ Although such interventions might reduce the adverse effects of prolonged arrest and attenuate any associations with cognitive outcomes, most participants in this cohort had short no‐flow (median: 0 minutes, Q25–Q75:0–2) and low‐flow times (median: 12 minutes, Q25–Q75:7–18). The influence of these unmeasured factors is therefore likely to be limited, although residual confounding cannot be ruled out.

A 21% dropout rate at the 6‐month follow‐up, most commonly due to participants reporting a lack of energy (Figure 1), may have influenced the results. Notably, 88% of those who dropped out had a MoCA score <26 at discharge, compared with 74% in the baseline sample (Table 2). This indicates a potential selection bias, which may have led to an overestimation of the observed cognitive improvement at follow‐up.

Before initiating the study, no formal sample size calculation was performed due to the absence of effect size estimates from comparable studies using MoCA as a cognitive outcome in OHCA survivors. The sample size was instead determined pragmatically, based on the number of eligible patients within the defined data collection period. This reflects both the exploratory nature of the study and the rarity of the target population.

The relationship between the duration of cardiac arrest and cognitive function is complex, and further research is required to better understand which factors influence cognitive impairments in OHCA survivors in order to devise targeted interventions aimed at improving long‐term cognitive function and quality of life for this population.

## CONCLUSIONS

This study examined the prevalence of cognitive impairment at hospital discharge and its progression over 6 months following cardiac arrest. Although only 26% of survivors had normal cognitive function at discharge, this increased to 67% after 6 months. Contrary to our hypothesis, no significant association was found between cardiac arrest duration and cognitive function assessed by MoCA.

## Sources of Funding

This work was funded by Aarhus University Hospital, Faculty of Health, Aarhus University, the Danish Health Confederation’s development and research fund (no. 2867) and the Danish Association of Occupational Therapists (no. FF2‐R104‐A1981).

## Disclosures

The authors declare there are no conflicts of interest.

## Supporting information

Data S1
