# Supplementary material for: Cognitive Recovery After Out‐of‐Hospital Cardiac Arrest: Insights Into Improvement Over 6 Months and the Role of Arrest Duration
Source: J Am Heart Assoc. 2025 Nov 3;14(21):e042637. doi: 10.1161/JAHA.125.042637 (PMC12684582; doi:10.1161/JAHA.125.042637)
Supplement: Supplementary file 1 — Data S1 [file JAH3-14-e042637-s001.pdf]

SUPPLEMENTAL MATERIAL

Logistic regression of the association between no-flow and low-flow (duration of cardiac arrest) and cognitive function measured with MoCA<26 six months after cardiac arrest, stratified by age

	Age <65, n=64			Age ≥65, n=76		
	OR	95% CI	p-value	OR	95% CI	p-value
No-flow	0.80	0.45; 1.42	0.457	1.10	0.44; 5.53	0.492
Low-flow	1.04	0.99; 1.08	0.086	1.02	0.96; 1.10	0.413
Time to ROSC	1.03	0.99; 1.08	0.092	1.02	0.97; 1.09	0.411

Adjusted for sex, comorbidities (yes/no), witnessed arrest, bystander CPR, the initial recorded rhythm and location of cardiac arrest.

Logistic regression of the association between no-flow and low-flow (duration of cardiac arrest) and cognitive function measured with MoCA<26 six months after cardiac arrest, stratified by the burden (number) of comorbidities

	0 comorbidity, n=40			1 comorbidity, n=72			2-4 comorbidities, n=23		
	OR	95% CI	p-value	OR	95% CI	p-value	OR	95% CI	P-value
No-flow	1.59	0.80; 3.14	0.186	0.70	0.38; 1.29	0.255	0.92	0.41; 2.08	0.290
Low-flow	1.07	0.99; 1.15	0.108	1.01	0.92; 1.09	0.964	1.02	0.90; 1.16	0.750
Time to ROSC	1.07	0.99; 1.02	0.090	0.99	0.92; 1.08	0.894	1.01	0.90; 1,16	0.779

Adjusted for age, sex, witnessed arrest, bystander CPR, the initial recorded rhythm and location of cardiac arrest.
